# The Effects of Tai Chi on Peripheral Somatosensation, Balance, and Fitness in Hispanic Older Adults with Type 2 Diabetes: A Pilot and Feasibility Study

**DOI:** 10.1155/2015/767213

**Published:** 2015-10-27

**Authors:** Elisabeth I. Cavegn, Jody L. Riskowski

**Affiliations:** ^1^University of Texas at El Paso, El Paso, TX 79968, USA; ^2^Institute for Allied Health Research, Glasgow Caledonian University, Glasgow G4 0BA, UK

## Abstract

Peripheral neuropathy and loss of somatosensation in older adults with type 2 diabetes can increase risk of falls and disability. In nondiabetic older adult population Tai Chi has been shown to enhance balance and fitness through improvements in somatosensation and neuromuscular control, and it is unclear if Tai Chi would elicit similar benefits in older adults with diabetes. Therefore, the purpose of this study was to investigate the effects of an 8-week, three-hour-per-week Tai Chi intervention on peripheral somatosensation in older adults with type 2 diabetes. Participants were eight Hispanic older adults with type 2 diabetes who participated in the Tai Chi intervention and a convenience sample of Hispanic older adults as a referent group. Baseline and postintervention assessments included ankle proprioception, foot tactile sense, plantar pressure distribution, balance, and fitness. After intervention, older adults with type 2 diabetes showed significant improvements in ankle proprioception and fitness and decreased plantar pressure in the forefoot, with no statistical effect noted in balance or tactile sensation. Study results suggest that Tai Chi may be beneficial for older adults with diabetes as it improves ankle proprioception; however, study findings need to be confirmed in a larger sample size randomized controlled trial.

## 1. Introduction

More than 220 million people worldwide have diabetes, and this number is expected to double by 2030 [1]. Of those with diabetes, approximately 90% is type 2 diabetes or non-insulin-dependent diabetes mellitus (NIDDM) in which the body does not produce enough insulin or utilizes the available insulin inefficiently [2]. NIDDM is often the result of uncontrolled metabolic syndrome, which is a cluster of interrelated risk factors including elevated blood pressure, high levels of blood lipids, elevated fasting glucose, and obesity [3]. The chronically elevated blood glucose level characteristic of NIDDM predisposes patients to long-term complications [4] and impairs physical functioning [5]. Key contributors to physical impairment in individuals with type 2 diabetes include uncontrolled hyperglycemia, reduced peripheral blood flow, weakened muscle strength, altered proprioception, and diminished motor coordination [5, 6].

Reduced blood flow and uncontrolled hyperglycemia can also lead to peripheral neuropathy or the progressive loss of nerve fibers. Peripheral neuropathy reduces foot sensation, muscle activation of the intrinsic foot muscles, and vasomotor control of the pedal circulation [7, 8]. While some people with neuropathy experience symptoms that manifest as burning sensation, sharp pain, numbness, and pain to normal touch, the majority have insidious symptoms, including the inability to feel, assess temperature, or sense painful stimuli. Loss of pedal protective sensation increases risk for microtrauma and hidden injury to the foot, which can cause infections, calluses, and foot ulceration [9, 10].

Although not all individuals with type 2 diabetes develop peripheral neuropathy, chronically elevated blood glucose level can also comprise sensorimotor receptors and balance control [11]. Altered sensorimotor signaling along with diminished peripheral sensation and postural instability [12] may explain why older adults with diabetes have a two- to threefold higher risk of falls and physical disability compared to nondiabetic elderly [13].

To reduce fall risk, older adults are encouraged to partake in exercise programs with resistance, endurance, balance, and flexibility training. Benefits of such programs include improved overall health and the preservation of independence [14, 15]. More recently, Tai Chi has been recognized as an effective alternative to traditional exercise programs for fall prevention [16] and to improve plantar sensation [17]. Tai Chi, an ancient Chinese martial art, incorporates elements of strength, balance, postural control, and concentration, and it is recognized as one of the most effective interventions for reducing falls in the older adult population [14–16]. A mechanism for improving fall risk through Tai Chi may be through the benefits of improved proprioception, particularly at the ankle joint, and through improved tactile sense [17–19].

While interest in Tai Chi's benefits is growing in the western world, the scientific literature showing benefits of Tai Chi in older adults with diabetes and peripheral sensory loss is unknown. With the growing prevalence of type 2 diabetes among older adults and the associated high fall risk, there is a need for appropriate treatment strategies and therapeutic interventions. Therefore, the purpose of this study was to investigate the effectiveness of Tai Chi exercise for improving peripheral somatosensation as measured by ankle proprioception (primary outcome) and foot tactile sense (secondary outcome). The hypothesis was that a short-term Tai Chi program would improve ankle proprioception and foot tactile sense as well as improving balance, coordination, and overall fitness.

## 2. Methods

The study was a single-arm Tai Chi intervention with a pre-/posttest within-subjects design during 2011. The intervention phase for the older adults with diabetes consisted of three one-hour sessions of Tai Chi per week for eight weeks (24 one-hour sessions). Baseline participant data were collected within one week prior to the intervention commencement, with postintervention data collected within one week following the conclusion of the intervention. Healthy older adults who did not have diabetes were also included for a referent population. All participants were informed of their rights as study participants and signed an informed consent approved by the University's Institutional Review Board.

### 2.1. Participants

Eight Hispanic older adults with type 2 diabetes were recruited by direct contact from educational classes at a local diabetes charity and from a waitlist for a university wellness program, which they did not commence until after the Tai Chi study ended. Study inclusion criteria were age 55 and 80 years and diagnosis of type 2 diabetes for more than five years. Exclusion criteria included engaging in moderate or strenuous exercise three months prior to the start of this study, inability to walk independently, chronic medical problems (other than diabetes) that limited physical activity participation, a body mass index (BMI) greater than 40 kg/m^2^, and cognitive impairment. Using similar inclusion/exclusion criteria, a convenience sample of eight Hispanic adults without diabetes was also recruited to serve as a referent population.

### 2.2. Tai Chi Intervention

Tai Chi lessons were taught by an experienced Tai Chi instructor who followed the classic Yang style (long form, first section), which emphasizes multidirectional weight shifting, awareness of body alignment, and coordination of movement. Additionally, guidance in correct breathing techniques was provided and integrated into the Tai Chi routine. Each session began with specific warm-up exercises for approximately 10 minutes, followed by 45 minutes of Tai Chi practice, and ended with approximately five minutes of breathing exercises. During the practice, previously learned forms were repeated prior to adding new forms. Furthermore, the instructor placed great emphasis on the correct execution of the movements, particularly on foot placement, weight shifts, and postural alignment. There were three sessions per week for 8 weeks (24 hours of instruction) with the instructor logging class attendance to evaluate adherence to the Tai Chi intervention. There was no set schedule participants had to follow, and participants were able to attend any of the Yang-style classes offered by the instructor at a time that was convenient to them. There was no home practice required for the study, and home practice was not monitored. There was no expectation or assessment regarding how much of the Tai Chi form they would remember through the 8-week course.

### 2.3. Outcome Measures

The primary outcome measure was ankle proprioception, with secondary measures of foot tactile and balance sense, plantar pressure distribution during standing, and fitness. All outcome measures were performed by a single trained evaluator who was not blind to the participant group.

#### 2.3.1. Ankle Proprioception

Proprioceptive measures were evaluated by determining the participants' ability to reproduce a target joint angle in a joint angle reproduction (JAR) test. A universal goniometer (Baseline, Chattanooga Group, Inc., Hixson, TN) was used for the JAR test. In this JAR test, from a neutral position (90° at the ankle joint), the ankle was moved slowly to either a target angle of 10° dorsiflexion or 10° plantarflexion from neutral, with this target angle held for five seconds. After returning the ankle to the neutral position, participants were asked to reproduce the target angle. Difference between the target and actual angle to the nearest ±0.5° was recorded. There were five trials for each ankle movement for a total of 10 trials per foot. Order of testing for the movements to dorsiflexion and plantarflexion was randomized, with all participants undergoing the same testing order. Mean JAR was calculated and presented as absolute error, reflecting the total number of degrees away from the target angle, and as relative error with positive and negative values representing the degrees of overshoot and undershoot, respectively [20].

#### 2.3.2. Foot Tactile Sense

**Figure 1:**
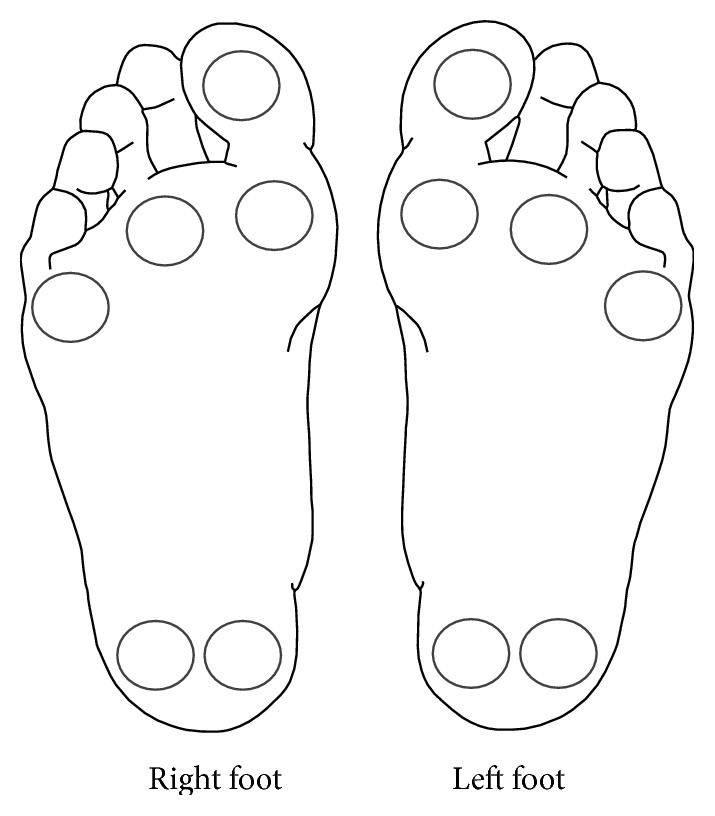
Tested sites of the Semmes-Weinstein monofilament assessment for peripheral neuropathy.

The Semmes-Weinstein monofilament test was used to measure plantar nerve sensitivity. For this study, a 5.07/10 g monofilament was used because detection denotes the threshold of “protective sensation” and the sensation necessary to prevent hidden injury [21]. Six pedal sites were tested on each foot (Figure 1). Each site was tested three times, with two “active” tests where the monofilament touched the skin and one “sham” where the monofilament did not touch the skin. Testing stimuli (active or sham) were randomly presented three times at each of the six sites, and participants were asked “do you feel it—yes or no?” Order of sham and active application and order of pedal site were consistent for all participants. Participants were classified as having peripheral neuropathy if two of the three applications at a site were answered incorrectly.

#### 2.3.3. Balance

Balance was assessed using center of pressure (COP) displacement in the anterior-posterior (A-P) and medial-lateral (M-L) direction during double-leg stance with eyes closed using the Tekscan MatScan (Tekscan, Inc., Boston MA, USA, capture rate: 100 Hz). The eyes-closed assessment was chosen as a means for reducing bias for variability in visual acuity between participants and for increasing the challenge of a bipedal balance test [22]. Participants stood in a self-selected stance with eyes closed and arms across the chest on the pressure mat for 30 seconds. Total sway area and A-P and M-L COP displacement were computed by using the sway analysis module (Tekscan, Inc., Boston MA, USA, v6.40).

#### 2.3.4. Plantar Pressure Distribution

From the balance data, plantar pressure for each foot was evaluated under the great toe, 1st, 3rd, and 5th metatarsal head (MTH), and heel. These sites were chosen because pressure changes from the heel to the forefoot in people with diabetes may present an early marker of peripheral neuropathy [23]. Crude and normalized (by body mass) mean peak plantar pressure was evaluated at each site.

#### 2.3.5. Senior Fitness Test (SFT)

The senior fitness test comprises six assessments: arm curls, chair stands, back scratch test (shoulder girdle flexibility), chair sit-and-reach (hamstring flexibility), eight-foot timed-up-and-go (TUG), and six-minute walk test, with this order of testing consistent for all participants [24]. Each component was explained and demonstrated, with participants having one familiarization trial to ensure the correct form before the recorded testing.

### 2.4. Data Analysis

All data were checked for normality using Shapiro-Wilk's test, and data were normalized through a log transformation, if necessary. Differences between the pre- and postintervention were recorded, and a one-sample *t*-test was used to determine if difference scores were significantly different from zero. A Mann-Whitney *U*-test was used to compare the nontransformed posttest data between participants with and without diabetes to evaluate differences between the two groups.

Data analyses were conducted using the statistical software package SPSS v18.0 (SPSS Inc., Chicago, IL). Notwithstanding problems associated with the small sample size and multiple comparison testing, an unadjusted significance level of 5% was used. Although the unadjusted *p* value may increase the likelihood of false positives, it is a valid means for pilot testing for hypothesis generating, as opposed to drawing definitive conclusions [25]. Given that the work was a pilot study, effect sizes using Cohen's *d* for the measures were also included, and these were calculated based on correlations between the pre- and postintervention values for Tai Chi participants [26, 27].

## 3. Results

There were no statistically significant differences in height, weight, and BMI between the referent and diabetic groups (*p* ≥ 0.208; Table 1). All participants with diabetes completed the Tai Chi intervention and no adverse events were observed. Mean Tai Chi attendance rate was 94.8% (range 100% [*n* = 2] to 87.5% [*n* = 1]). Reasons for the absences were sickness, family obligations, weather, work schedule, and jury duty.

At baseline, absolute ankle joint angle reproduction (JAR), a measure of ankle proprioception between those with and without diabetes (referent), was significantly different in dorsiflexion and plantarflexion (Table 2). Following the Tai Chi intervention, significant improvements in ankle proprioception were noted for the participants with diabetes, with the JAR scores in ankle proprioception becoming more like the referent population over the 8-week intervention. Effect sizes using Cohen's *d* for ankle proprioception from pre- to postintervention were large and ranged from 1.211 to 1.837.

One participant with diabetes presented with peripheral neuropathy (lateral and medial aspects of both heels) at baseline. Following the Tai Chi intervention, there was no change in the diabetic group's peripheral neuropathy scores, as the same individual showed no signs of improvement in the neuropathic aspects of the heels. Due to the limited differences between the groups and the lack of change over the course of the intervention, no statistical tests were performed.

No significant differences for total peak plantar pressure or peak plantar pressure normalized to body mass were found among the five assessment sites between the referent and diabetic groups during quiet standing at baseline (Table 3). A reduction in peak pressure at the right and left 1st MTH was observed for the diabetic group following the intervention, but these changes in peak pressure were not statistically significant nor where they statistically different from the referent. Effect sizes using Cohen's *d* for the plantar pressure measures for effect of the intervention ranged from small to moderate (0.000–0.465), with mean plantar pressure Cohen's *d* of 0.280 and 0.262 for the nonnormalized and normalized plantar pressures, respectively.

After intervention, individuals with diabetes did not have statistically significant changes in sway area or A-P and M-L COP displacement, and there were no differences between those with and without diabetes in these balance variables (Table 4). Effect sizes using Cohen's *d* for the balance measures were small to moderate, with the medial-lateral sway showing the largest effect at 0.597.

From baseline to postintervention, individuals with diabetes significantly improved arm strength, as measured by the arm curls, and flexibility, as measured by the back scratch test (shoulder flexibility) and chair sit-and-reach (hamstring flexibility; Table 5). Following the intervention, TUG time and 6MWT distance were also significantly improved. Finally, baseline significant differences for the left back scratch and 6MWT at baseline between the referent and individuals with diabetes were lost through the intervention period. Excluding the back scratch measure (mean Cohen's *d* of 0.299), effect sizes using Cohen's *d* were moderate to large for the physical fitness measures and ranged from 0.497 to 1.659.

## 4. Discussion

The purpose of this study was to evaluate the effects of eight weeks of Tai Chi practice on balance and peripheral somatosensation, including both ankle proprioception (primary outcome) and foot tactile sense, in older adults with type 2 diabetes. The major finding of this study was that during an 8-week Tai Chi ankle proprioception in older adults with type 2 diabetes was enhanced. The results also showed that despite a gain in ankle proprioception, there was no significant change in balance (i.e., sway area and sway distance). Further, after an 8-week moderate-intensity, low-impact practice of Tai Chi program, participants had higher senior fitness test scores. Lastly, given the high adherence (95%) and retention (100%), these results suggest that Tai Chi may be an agreeable exercise activity for Hispanic older adults with diabetes. These findings suggest that 8-week Tai Chi may positively affect ankle proprioception and fitness in older adults with type 2 diabetes.

This study assessed both foot tactile sense and ankle proprioception to examine the effects on balance and neuropathy. Because only two participants presented with minor loss of tactile sensation, the potential effect of Tai Chi on this variable could not be evaluated; however, other studies have noted improvements in plantar tactile sense over a 24-week Tai Chi intervention [17]. With respect to ankle proprioception, scientists have suspected that it may be impaired in individuals with diabetes, but information concerning the morphological effects of diabetes on proprioceptive receptors is limited. This study showed that proprioception is impaired in people with diabetes but can be significantly improved with regular Tai Chi practice. The comparison with the referent group revealed that ankle flexion was to a greater extent affected by loss of proprioceptive sensation than ankle extension, and after an eight-week Tai Chi intervention ankle proprioception in those with diabetes became more similar to that of the referent group.

There are several theories that may explain Tai Chi's positive influence on ankle proprioception. First, the deliberate and repeated practice of complex movement patterns associated with Tai Chi may intensify the sensitivity of the proprioceptors, increasing reliance on afferent input and stimulating resensitization of peripheral sensory receptors [19]. Second, the continuous shifting of weight through the Tai Chi practice may enhance circulation of blood in the lower extremities [28], thereby providing necessary oxygen and energy to sensory receptors. Between resensitization of the peripheral sensory receptors and nourishment of the lower extremity tissues, improved ankle proprioception may occur. Though directionally opposite, this view may be supported by finding that prolonged ischemia induced by a tourniquet applied above the ankle reduced passive joint position sense in healthy participants [8]. Lastly, Tai Chi may lower glycated haemoglobin (HbA1c) levels [29], which could contribute to increased ankle range of motion and improved proprioception. This theory would be indirectly supported by findings that long-term exposure to high glucose levels can stiffen the ligaments and tendons in the lower extremities through a process known as glycosylation [2], leading to compromised joint mobility and increased ankle stiffness [30], which is associated with loss of proprioception [31].

Similar to the work of Acharya and colleagues [2], a tendency for anterior plantar pressure displacement in people with diabetes was also observed in this study relative to those without diabetes. Those with diabetes had higher peak pressure in the great toes and the 1st MTH than the referent group, whereas the pressure in the heels was lower. The cause of elevated forefoot plantar pressure in people with diabetes may be a combination of decreased foot and ankle flexibility and altered proprioception. Lack of range of motion in the ankle joint requires an extra force on the forefoot, leading to more pressure in the forefoot than in the heel [2]. Impaired proprioception creates an imbalance between the long flexors and extensors of the toes, leading to structural changes that increase foot supination and pressure under the 4th and 5th MTH [32]. Foot pressure changes in diabetic patients without any clinical evidence of neuropathy may present an early marker of peripheral neuropathy [23].

Despite the study results showing nonsignificant changes in plantar pressure, other studies suggest that regular Tai Chi practice may have the potential to distribute the plantar pressure more evenly across a person's foot suggesting implications for the prevention of peripheral neuropathy, as Tai Chi appears to target factors responsible for the foot pressure abnormalities that precede peripheral neuropathy [33]. To more fully evaluate this relationship between somatosensation, plantar pressure, and Tai Chi exercise, future work should examine the muscle activation patterns of older adults with and without peripheral neuropathy for both standing and walking and in more challenging tasks, such as obstacle crossing. Evaluating the plantar pressures along with a more in-depth analysis of the kinematic and kinetic adaptations that occur at the foot and ankle may help explain how changes that arise with Tai Chi exercise affect lower extremity functioning in this population.

Moreover, loss of balance in the elderly is more likely to occur during dynamic activities such as walking or maneuvering obstacles; research suggests that older adults with diabetic neuropathy have poorer postural control during quiet standing with eyes closed compared to their nondiabetic counterparts [34]. Results from this study showed no significant differences or improvements in sway area or COP displacement in the A-P and M-L direction between the referent and diabetic groups at baseline and after intervention. One explanation could be that an 8-week Tai Chi intervention (24 hours of practice) may not have provided sufficient training, as research has shown that improvements in sway become most evident after more than 40 hours of Tai Chi practice [35]. Further, because Tai Chi involves controlled movements and changes of body position in different directions, a test assessing functional balance, such as stepping over an obstacle, may be a better means to evaluate older adults' balance control, fall risk, and movement efficiency [33, 36].

Tai Chi is generally associated with favorable effects on cardiorespiratory fitness, flexibility, and lower extremity strength in older people [18, 37], and this study shows similar improvements in fitness. The large improvement observed in previously sedentary people following an eight-week, low-to-moderate-intensity Tai Chi intervention may be a combination of different factors rather than an increase in cardiovascular endurance alone [18, 37]. It is possible that improved plantar pressure distribution together with enhanced proprioception and ankle flexibility increased participants' walking confidence and efficiency. Further, gains in walking, regardless of the mechanism, could have led to increased physical activity outside the Tai Chi intervention. In the future, physical activity levels before, during, and following the Tai Chi intervention should be assessed to evaluate if there are changes in daily physical activity. Nonetheless, Tai Chi's effectiveness for improving overall fitness appears to be in its slow, purposeful movement patterns emphasizing body and trunk rotation, postural alignment, and coordination of the upper extremities [18, 37]. These movements, executed in a semisquat position, are thought to help mobilize the whole body, improve flexibility and leg strength, and increase cardiovascular endurance [38].

### 4.1. Strengths and Limitations

There are several strengths and limitations to this study. First, the study was a quasiexperimental pre-/posttest single-arm pilot study without a control and/or traditional exercise group to compare results against. Therefore, the improvements experienced by the older adults with diabetes may be related to increases in physical activity in general, not attributable to the Tai Chi study. However in prior studies of knee proprioception changes through Tai Chi compared to a control population of no Tai Chi, knee proprioception only improved in the Tai Chi group [39, 40]. Future work should evaluate the health benefits of various modalities of physical activity in order to determine what activities yield the strongest gains in balance and peripheral somatosensation. Second, as a pilot and feasibility study, the small sample size limits generalizability to a broader diabetic population. The study was powered to detect significant changes but to provide evidence for sample size estimates for future studies in this domain. Moreover, the study was not blinded with all data collected from a single researcher; however, data were evaluated or analyzed until after the completion of the study in order to reduce the bias that can be present in unblinded studies. Further, there was only one Tai Chi instructor involved in providing the Tai Chi sessions, which means it is unclear if the session adherence and gains are a result from enjoying the Tai Chi practice or a result of enjoying time with the instructor. Lastly, this study only explored the neurologic components of proprioception and tactile sense; however, both cardiovascular and neurologic components are manifestations of the mechanisms being at play in the diabetic foot. While this study did not see a change in neuropathy (only one participant had neuropathy), it could be that Tai Chi also affected the vascular components, such as peripheral artery disease. Future work should thus investigate if measures of micro- or macrovascular disease change in older adults with type 2 diabetes with Tai Chi practice, which may help elucidate the health benefits of Tai Chi for this population. A strength of the study included the measures regarding mechanisms for improved balance control and the measures of proprioception and foot tactile sense. Findings from this pilot study that suggest an 8-week Tai Chi program can enhance ankle proprioception, which may have important implications for the diabetic population, as poor ankle proprioception may adversely affect fall risk [41] and adversely affect plantar pressure distribution [42] leading to an increased risk of foot ulceration [43]. Moreover, as nonwhite racial and non-Hispanic ethnic communities in the United States are less inclined to partake in mind/body practices, such as yoga and Tai Chi [44], despite the increased risk of poor health [45, 46], this study with its 95% adherence rate over 24 sessions shows that nontraditional exercise modalities can be well received in Hispanic community.

## 5. Conclusion

With the growing prevalence of type 2 diabetes in older adults, understanding and improving health and physical function in this population are of paramount importance, and Tai Chi may be an effective therapeutic modality in this population. Although exploratory in nature, results from this study suggest that Tai Chi may positively affect lower extremity health through improving ankle proprioception and through redistributing the plantar pressure away from the ulcer-prone sites of the forefoot. These mechanisms have been shown to improve automatic balance correcting responses, and this study along with prior work [47, 48] suggests that Tai Chi may be an effective activity to reduce the fall risk in older adults with diabetes. Furthermore, Tai Chi may be an exercise intervention of choice for maintaining and improving physical fitness of older adults with type 2 diabetes. Lastly, adherence (95%) and retention (100%) were high, suggesting that Hispanic older adult populations with diabetes may find nontraditional exercise modalities, such as Tai Chi, as an amenable activity. However, the findings of this study should be interpreted with caution. More controlled and randomized studies with larger sample sizes are required to determine whether the trends shown in this study are truly applicable to this population.

## Figures and Tables

**Table 1:** Participant characteristics. Data are presented as means ± standard deviation, unless otherwise noted.

	Referent group	Diabetic group
	*N* = 8	*N* = 8
Number of women (%)	6 (75)	6 (75)
Age, years	63.8 ± 5.7	65.5 ± 7.4
Height, cm	163.4 ± 7.5	162.0 ± 9.8
Body mass, kg	76.3 ± 12.0	82.6 ± 13.9
Body mass index (BMI), kg/m^2^	28.6 ± 3.8	31.5 ± 5.2
Length of diabetes diagnoses, years	—	18.63 ± 9.21

**Table 2:** Baseline and postintervention proprioception of patients with diabetes as well as referent data. Data are presented as means ± standard deviation.

	Referent group	Diabetic group baseline	Diabetic group after intervention	Intervention group Cohen's *d*
Relative mean right ankle (°)				
Dorsiflexion	0.85 ± 1.36	4.68 ± 2.75^†^	2.10 ± 1.75^*∗*^	1.480
Plantarflexion	1.00 ± 0.98	2.20 ± 1.59	1.08 ± 0.76^*∗*^	1.211
Absolute mean right ankle (°)				
Dorsiflexion	1.80 ± 0.70	4.98 ± 2.41^†^	2.30 ± 1.64^*∗*^	1.738
Plantarflexion	1.45 ± 0.45	2.65 ± 1.24^†^	1.30 ± 0.76^*∗*^	1.837

Relative mean left ankle (°)				
Dorsiflexion	1.45 ± 0.64	3.95 ± 3.33	1.83 ± 1.78^*∗*^	1.173
Plantarflexion	0.58 ± 1.10	2.88 ± 2.25	1.00 ± 0.99	1.609
Absolute mean left ankle (°)				
Dorsiflexion	1.60 ± 0.59	4.45 ± 2.85^†^	1.93 ± 1.74^*∗*^	1.352
Plantarflexion	1.63 ± 0.46	3.08 ± 2.13^†^	1.30 ± 0.83^*∗*^	1.527

^*∗*^Significant change between pre- and posttest for diabetic group (*p* ≤ 0.05).

^†^Significant difference between referent group and diabetic group at baseline (*p* ≤ 0.05). No significant differences remained at posttest between groups.

**Table 3:** Plantar pressure distribution during two-foot self-selected standing balance. Data are presented as means ± standard deviation. Normalized data is normalized to participant's body mass. MTH = metatarsal head.

Pressure region	Referent group	Diabetic group baseline	Diabetic group after intervention	Intervention group Cohen's *d*
Total peak plantar pressure (kPa/cm^2^)				
Peak plantar pressure right foot				
Great toe	26.39 ± 15.86	48.63 ± 39.38	47.84 ± 36.83	0.121
1st MTH	61.00 ± 26.26	108.82 ± 56.62	90.38 ± 53.01^*∗*^	0.415
3rd MTH	93.00 ± 26.79	96.50 ± 56.42	89.25 ± 36.18	0.277
5th MTH	75.00 ± 51.79	68.75 ± 43.31	58.32 ± 36.39	0.127
Heel	160.00 ± 51.43	118.88 ± 52.20	117.38 ± 47.94	
Peak plantar pressure left foot				
Great toe	39.00 ± 19.29	47.38 ± 55.71	32.63 ± 31.15	0.421
1st MTH	56.00 ± 24.40	112.38 ± 77.77	103.38 ± 89.62	0.285
3rd MTH	93.00 ± 33.77	98.00 ± 49.23	89.00 ± 46.13	0.277
5th MTH	77.00 ± 42.26	59.13 ± 33.31	57.38 ± 26.48	0.158
Heel	157.00 ± 66.52	121.38 ± 48.31	119.75 ± 36.88	0.156

Normalized peak plantar pressure (kPa/cm^2^/kg)				
Peak plantar pressure right foot				
Great toe	0.33 ± 0.21	0.54 ± 0.34	0.57 ± 0.39	0.204
1st MTH	0.89 ± 0.38	1.43 ± 1.10	1.28 ± 1.01	0.278
3rd MTH	1.13 ± 0.28	1.20 ± 0.78	1.10 ± 0.52	0.287
5th MTH	1.00 ± 0.62	0.82 ± 0.49	0.70 ± 0.41	0.439
Heel	1.98 ± 0.59	1.39 ± 0.60	1.38 ± 0.61	0.111
Peak plantar pressure left foot				
Great toe	0.47 ± 0.25	0.52 ± 0.55	0.39 ± 0.37	0.465
1st MTH	0.76 ± 0.34	1.53 ± 1.52	1.46 ± 1.98	0.060
3rd MTH	1.11 ± 0.27	1.17 ± 0.65	1.06 ± 0.62	0.255
5th MTH	0.84 ± 0.19	0.68 ± 0.34	0.68 ± 0.26	0.000
Heel	2.05 ± 0.82	1.42 ± 0.50	1.42 ± 0.40	0.000

^*∗*^Significant change between pre- and posttest for diabetic group (*p* ≤ 0.05).

**Table 4:** Balance and center of pressure (COP) sway distance at baseline and after intervention for the individuals with diabetes compared to a referent population of older adults. Data are presented as means ± standard deviation.

	Referent group	Diabetic group baseline	Diabetic group after intervention	Intervention group Cohen's *d*
Sway area (cm^2^)	1.53 ± 1.10	2.10 ± 1.73	1.79 ± 1.22	0.267
COP anterior-posterior (cm)	2.35 ± 0.79	3.00 ± 0.97	2.94 ± 1.29	0.071
COP medial-lateral (cm)	1.51 ± 0.77	1.55 ± 0.64	1.31 ± 0.37	0.593

**Table 5:** Senior fitness test at baseline and after intervention for the individuals with diabetes compared to a referent population of older adults. Data are presented as means ± standard deviation.

	Referent group	Diabetic group baseline	Diabetic group after intervention	Intervention group Cohen's *d*
Arm curls (repetitions)				
Right arm	22.50 ± 4.57	21.75 ± 3.73	26.88 ± 4.39^*∗*^	1.659
Left arm	22.38 ± 4.50	22.0 ± 4.54	26.25 ± 4.43^*∗*^	1.223
Chair test (repetitions)	12.63 ± 2.92	11.75 ± 3.58	14.38 ± 4.81^*∗*^	0.796
Back scratch (cm)				
Right side	−7.77 ± 16.75	−21.56 ± 14.21	−18.80 ± 12.87^*∗*^	0.243
Left side	−12.33 ± 9.58	−27.48 ± 15.87^†^	−23.33 ± 14.31^*∗*^	0.354
Chair sit-and-reach (cm)				
Right leg extended	1.74 ± 9.92	−6.23 ± 8.34	0.04 ± 5.28^*∗*^	1.187
Left leg extended	2.84 ± 11.27	−6.48 ± 7.98	−0.48 ± 5.00^*∗*^	1.233
8-foot up-and-go (s)	5.81 ± 0.81	7.78 ± 2.60	6.84 ± 2.22^*∗*^	0.520
6-minute walk test (m)	554.35 ± 93.21	412.77 ± 126.76^†^	462.61 ± 123.32^*∗*^	0.497

^*∗*^Significant change between pre- and posttest for diabetic group (*p* ≤ 0.05).

^†^Significant difference between referent group and diabetic group at baseline (*p* ≤ 0.046). No significant differences remained at posttest.
